# Impact of High-Dose Rifampicin on Linezolid Pharmacokinetics in Tuberculous Meningitis

**DOI:** 10.1093/ofid/ofag154

**Published:** 2026-03-27

**Authors:** Dvijen Purohit, Rob van Wijk, Paddy Kafeero, Freddie Kibengo, Matthew Zimmerman, Veronique D'artois, Felicia C Chow, Rada M Savic

**Affiliations:** Department of Bioengineering and Therapeutic Sciences, University of California, SanFrancisco, California, USA; UCSF Center for Tuberculosis, University of California, SanFrancisco, California, USA; Department of Bioengineering and Therapeutic Sciences, University of California, SanFrancisco, California, USA; UCSF Center for Tuberculosis, University of California, SanFrancisco, California, USA; Medical Research Council/Uganda Virus Research Institute and London School of Hygiene and Tropical Medicine Uganda Research Unit, Entebbe, Uganda; Medical Research Council/Uganda Virus Research Institute and London School of Hygiene and Tropical Medicine Uganda Research Unit, Entebbe, Uganda; Center for Discovery and Innovation, Hackensack Meridian Health, Nutley, New Jersey, USA; Center for Discovery and Innovation, Hackensack Meridian Health, Nutley, New Jersey, USA; UCSF Center for Tuberculosis, University of California, SanFrancisco, California, USA; Department of Neurology and Medicine (Infectious Diseases) and Weill Institute for Neurosciences, University of California, SanFrancisco, California, USA; Department of Bioengineering and Therapeutic Sciences, University of California, SanFrancisco, California, USA; UCSF Center for Tuberculosis, University of California, SanFrancisco, California, USA

**Keywords:** CSF, drug interaction, TB meningitis, tuberculosis, high-dose rifampicin, linezolid, pharmacokinetics

## Abstract

**Background:**

Tuberculous meningitis (TBM), a severe form of extrapulmonary tuberculosis, requires sustained therapeutic drug concentrations in the central nervous system (CNS). Linezolid is a promising treatment for TBM due to excellent CNS penetration and bactericidal activity against *Mycobacterium tuberculosis*. However, co-administration with rifampicin may alter linezolid pharmacokinetics (PK), potentially reducing efficacy due to drug–drug interactions.

**Methods:**

We utilized data from the Adjunctive Linezolid for the Treatment of TubERculous Meningitis trial, a phase II study evaluating high-dose (35 mg/kg) versus standard-dose (10 mg/kg) rifampicin, with or without linezolid, in adults with TBM. Plasma sampling occurred on days 1 (dense) and 14 (sparse) with cerebrospinal fluid (CSF) sampling. Population PK modeling and simulation were employed to characterize linezolid disposition in plasma and CSF.

**Results:**

Eighteen participants contributed 73 plasma and 30 CSF samples to the analysis. Plasma concentrations were best described by a 1-compartment model with linear absorption and clearance, linked to a CSF compartment. High-dose rifampicin increased linezolid clearance by 34.2%, reducing systemic and CNS exposure. Simulations demonstrated that 1200 mg once-daily dosing failed to maintain therapeutic plasma and CSF linezolid concentrations when co-administered with high-dose rifampicin. In contrast, 600 mg twice-daily achieved adequate linezolid exposures even with higher rifampicin doses.

**Conclusions:**

High-dose rifampicin significantly increases linezolid clearance, reducing plasma and CSF drug levels. However, twice-daily linezolid may mitigate this effect and maintain therapeutic concentrations. These findings underscore the importance of optimizing linezolid dosing when used with rifampicin in TBM and support evaluation in larger studies to guide treatment strategies.

Tuberculous meningitis (TBM) is the most severe and life-threatening manifestation of extrapulmonary tuberculosis, with high mortality rates and significant risk of severe neurological complications [1]. One factor that may contribute to poor outcomes in TBM is the failure of existing therapeutic regimens adapted from the treatment of pulmonary tuberculosis (TB) to achieve adequate drug concentrations in the central nervous system (CNS). To effectively target TBM, drugs must overcome multiple biological barriers, including the blood–brain barrier (BBB) and blood–cerebrospinal fluid (CSF) barrier, which serve not only as physical barriers but also as metabolic barriers containing numerous enzymes that can inactivate drugs and toxins before they enter the CNS [2]. In addition to penetrating these barriers, drugs must maintain bactericidal concentrations within the CNS for a sufficient duration [3]. Moreover, TBM-related changes in barrier permeability and protein concentrations can influence drug distribution, presenting significant therapeutic challenges for achieving effective CNS drug levels [4]. Linezolid is an oxazolidinone antibiotic with good CNS penetration that inhibits bacterial protein synthesis and has demonstrated early bactericidal activity against *Mycobacterium tuberculosis* in pulmonary TB, making it a promising candidate in the treatment of TBM [5]. Adjunctive linezolid, given early during TBM treatment, may enhance bactericidal activity against *M. tuberculosis* during its rapid growth phase while minimizing toxicities more common with prolonged use. In observational studies of adults and children with TBM, linezolid has been associated with improved neurological outcomes and reduced mortality [6, 7]. Motivated by these favorable observations, linezolid is under investigation in multiple clinical trials as part of intensified antibiotic regimens for the treatment of TBM [8, 9].

The pharmacokinetics (PK) of linezolid in TBM may be affected by disease-related factors, including patient characteristics such as body size or sex, and disease-specific features such as BBB integrity, which together could impact its safety and efficacy given linezolid's narrow therapeutic window. Furthermore, the current standard of care for the treatment of drug-susceptible TBM is a combination regimen that includes rifampicin among other drugs, which poses its own unique set of challenges. Rifampicin is a potent inducer of the cytochrome P450 (CYP) system, which is responsible for the metabolism of many drugs, along with transporters such as P-glycoprotein or ATP-binding cassette subfamily B member 1 (ABCB1), which is involved in cellular efflux of xenobiotics and limiting the entry of drugs into the CNS from the BBB and absorption from the gastrointestinal tract [10, 11]. Co-administration of linezolid and rifampicin in TBM therapy may result in clinically significant drug interactions, potentially leading to suboptimal CSF concentrations of linezolid and increasing the risks of treatment failure and development of resistance [12, 13].

This analysis investigated the PK of linezolid in both the plasma and CSF of Ugandan adults with confirmed or suspected TBM. We examined the influence of high-dose compared with standard-dose rifampicin on linezolid PK and, specifically, on the rifampicin–linezolid drug–drug interaction; evaluated how various covariates affected linezolid disposition in plasma and CSF; and performed PK simulations to analyze exposure and inform optimized dosing strategies.

## METHODS

### Patient Population, Setting, and Study Design

This analysis was part of the ALTER (Adjunctive Linezolid for the Treatment of TubERculous Meningitis) trial (NCT04021121), a phase II randomized, open-label trial of high- or standard-dose rifampicin with or without linezolid for the first 4 weeks of treatment for definite or suspected TBM in adults cared for at Masaka Regional Referral Hospital in Masaka, Uganda, between 10 September 2021 and 7 June 2023, as described previously [9]. Randomization was stratified by HIV status and stage of disease (grade 1 vs grades 2 and 3), as defined by the modified British Medical Research Council (BMRC) criteria.

### Treatment

Participants were randomized to linezolid 1200 mg daily versus no linezolid for the first 4 weeks of therapy with high (35 mg/kg) or standard (10 mg/kg) dose rifampicin (Supplementary Table 1). Although the study protocol specified a once-daily dose of 1200 mg of linezolid, the first 7 participants randomized to linezolid inadvertently received 600 mg twice daily. Additionally, all participants received standard treatment for TBM with isoniazid (5 mg/kg/day), pyrazinamide (25 mg/kg/day), ethambutol (20 mg/kg/day), corticosteroids (dexamethasone 0.4 mg/kg/day for 1 week, 0.3 mg/kg/day for 1 week, followed by a prednisone taper over 6 weeks), and vitamin B6 (50 mg daily). After the initial 4 weeks of therapy, participants in the high-dose rifampicin group transitioned to standard-dose oral rifampicin (10 mg/kg/day), in accordance with national guidelines.

### Ethics

Written informed consent was obtained from participants. In cases of diminished capacity, consent was obtained from a designated surrogate caregiver or next of kin. Participants who regained capacity were re-consented for continued participation, with the option to withdraw. Ethics approval for the study was granted by the relevant institutional review board, ethics committees, and other national regulatory authorities.

### Pharmacokinetic Sampling

During the study period, CSF sampling via lumbar puncture (LP) was conducted at entry, with intensive plasma sampling at 0, 2, 4, and 8 hours postdose. A second CSF sampling occurred on day 14, along with sparse plasma sampling at a single time point. An optional LP was available at day 28 (week 4) ± 2 days for rifampicin and linezolid PK analysis for participants who were no longer hospitalized. For population analysis of the full CSF time curve while considering the invasive nature of the sampling procedure, participants were randomized to 1 of 3 CSF sampling intervals: early (0–2 hours), medium (2–6 hours), or late (6–8 hours) after drug administration.

Linezolid concentrations were quantified using a previously validated liquid chromatography–tandem mass spectrometry (LC-MS/MS) [14]. Samples were analyzed on a Sciex Applied Biosystems API 6500 + triple-quadrupole mass spectrometer paired with a Shimadzu Nexera X2 UHPLC system at the Center for Discovery and Innovation, Hackensack Meridian Health. Chromatographic separation was achieved on an Agilent Zorbax SB-C8 column (2.1 × 30 mm; particle size, 3.5 μm) using a reverse-phase gradient. The mobile phases consisted of 0.1% formic acid in Milli-Q water (A) and 0.1% formic acid in acetonitrile (B). Quantification was conducted in electrospray positive-ionization mode using multiple reaction monitoring of a parent → daughter transition of 338.20 → 296.00 for linezolid and internal standard linezolid-d3 (341.20/297.20) Analytical runs were considered acceptable when quality control samples were within 20% of their nominal values. Data were processed using Analyst software version 1.5.1 (Applied Biosystems Sciex).

### Pharmacokinetic Modeling

Plasma and CSF concentrations were analyzed by nonlinear mixed-effects modeling. Structural model development on plasma and subsequent sequential CSF concentration was followed by stochastic model development, inclusion of interindividual variability on structural parameters, and residual unexplained variability. For the structural model, 1- and 2-compartment models were tested as well as zero- and first-order absorption, and linear and nonlinear elimination. To characterize CSF concentrations, a partition coefficient and transfer rate constant to the CSF compartment were estimated, first with final plasma PK parameters fixed and simultaneously estimated for the final model. The transfer rate constant was fixed at a high value when insufficient information was available to estimate a possible delay between plasma and CSF. Interindividual parameters, assuming a log-normal distribution, were tested on all structural PK parameters. Residual unexplained variability, assuming a normal distribution, was tested in an additive, proportional, and combined additive and proportional manner on plasma and CSF combined and separately.

#### Covariates

Participant age, sex, weight, baseline Glasgow Coma Scale (GCS), GCS at week 1, GCS at week 4, renal function (assessed by calculating estimated glomerular filtration rate [eGFR] using the Cockroft–Gault equation from serum creatinine levels taken at entry), CSF cell count and protein, treatment arm (high- or standard-dose rifampicin), and absolute dose of rifampicin were considered as potential covariates on all structural plasma and CSF PK parameters in the analysis, including all scientifically plausible covariate–parameter relationships relevant to linezolid. A stepwise covariate model-building procedure was employed using Perl Speaks NONMEM (PsN) [15], following a forward inclusion (*P* < .05) and backward deletion (*P* < .01) approach to incorporate and retain covariates.

#### Model Selection and Evaluation

The selection of the model was conducted using a likelihood ratio test for nested models, applying a significance threshold of *P* < .05 for accepting any extensions to the model, assuming a χ^2^ distribution. To evaluate model performance, prediction-corrected and stratified visual predictive checks were employed [15], simulating 500 datasets that mirrored the original dataset's characteristics. The precision of parameter estimates was indicated by relative standard errors derived from the model. The PK analysis utilized the first-order conditional estimation method with interaction, performed using NONMEM version 7.4.3 [16], and incorporated features available in PsN. RStudio was used for graphical analysis (RStudio Team, 2022. RStudio: Integrated Development Environment for R. RStudio, PBC, Boston, MA, United States).

#### Pharmacokinetic/Pharmacodynamic Targets

We predicted secondary linezolid PK parameters, including percentage of time that plasma and CSF concentrations were above a pharmacodynamic breakpoint. Using this approach, we characterized the dynamic uptake of linezolid in the CSF when administered with high- and standard-dose rifampicin over 4 weeks. While pharmacodynamic breakpoints for linezolid against TB are not well established, we used a target value for minimum inhibitory concentration (MIC) of 1.0 μg/mL [17].

#### Simulations

Following finalization of the PK model, Monte Carlo simulations were performed using the demographic data of trial participants (500 trials). For each participant, exposure profiles for 1200 mg of linezolid, administered either once daily or as 2 divided doses, were simulated. These simulations were performed for each daily dose of rifampicin, assuming full adherence to represent the optimal treatment scenario (ie, treatment completion). Simulated plasma and CSF exposures were analyzed to assess the proportion of participants achieving PK/pharmacodynamic targets.

#### Statistical Analyses

Clinical and demographic characteristics were summarized using descriptive statistics. Continuous variables were presented as medians with interquartile ranges, while categorical variables were reported as frequencies and percentages. Data were analyzed using RStudio (RStudio Team, 2022).

## RESULTS

A total of 20 participants were randomized to linezolid. Two participants exhibited detectable linezolid levels prior to the first dose, suggesting discrepancies in either sampling or dosing records; these individuals were excluded. Of the remaining 18 participants in the analysis, the PK sampling included in the model spanned from the start of the study to 2 weeks, incorporating 103 total observations (Supplementary Table 2). Sparse sampling from nonhospitalized patients on day 28 was excluded due to the inability to reliably verify the timing of medication administration. All but 1 participant had CSF collected on day 1, and 13 participants underwent CSF sampling on day 14. The number of participants randomized to the early (0–2 hours), medium (2–6 hours), and late (6–8 hours) CSF sampling intervals was 5, 6, and 7, respectively. The median age of participants was 38.5 years (range, 26–72 years), and the median weight on the first day of PK sampling was 50 kg (range, 34–69 kg). All participants included in the analysis were living with HIV. Six were on antiretroviral therapy, with 1 participant on a regimen of tenofovir, lamivudine, and efavirenz, and the rest on tenofovir, lamivudine, and dolutegravir. Table 1 shows demographic and clinical characteristics of the participants. Twelve participants included in the analysis received 1200 mg of linezolid once daily; 6 participants received linezolid 600 mg twice daily.

**Table 1.** Demographic and Clinical Characteristics

Characteristics	All Participants (n = 18)	High-Dose Rifampicin (n = 8)	Standard-Dose Rifampicin (n = 10)
Age (y), median (IQR)	38.5 (33.3–42.8)	39.5 (37.5–42.5)	34.50 (33.0–42.8)
Female sex, n (%)	9 (50)	2 (25)	7 (70)
Patients with HIV infection, n (%)	18 (100)	8 (100)	10 (100)
Serum creatinine (mg/dL), median (IQR)	0.72 (0.56–0.75)	0.79 (0.64–0.87)	0.67 (0.56–0.77)
Medical Research Council Grade, n (%)			
Grade I	5 (27.8)	2 (25)	3 (30)
Grade II	9 (50)	4 (50)	5 (50)
Grade III	4 (22.2)	2 (25)	2 (20)
TBM case definition, n (%)			…
Definite	5 (27.8)	2 (25)	3 (30)
Probable	12 (66.7)	6 (75)	6 (60)
Possible	1 (5.6)	0 (0)	1 (10)

Abbreviations: IQR, interquartile range; TBM, tuberculous meningitis.

### Pharmacokinetic Model

**Figure 1.**
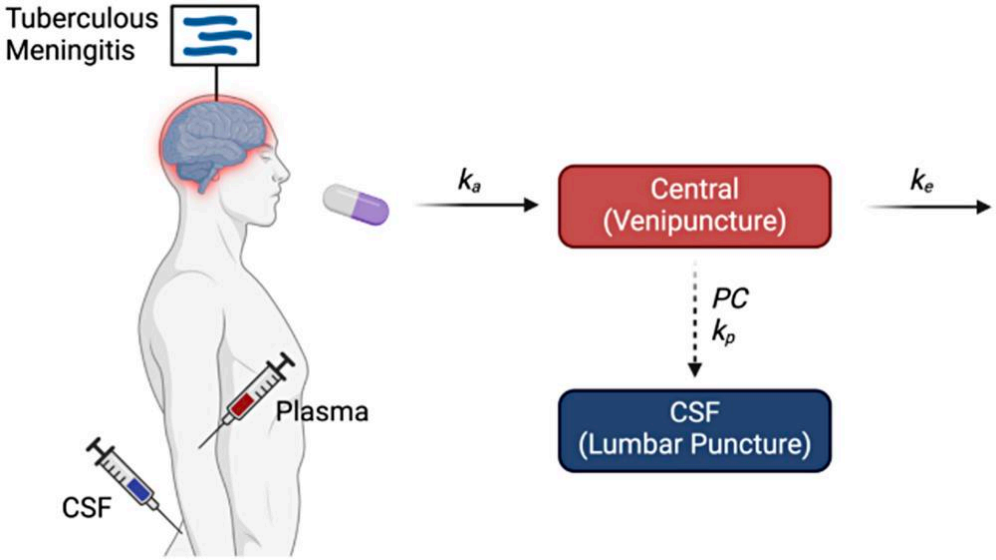
Final model structure: One-compartment model with linear absorption and clearance linked to the cerebrospinal fluid (CSF) compartment. *k*_a_ is the absorption rate constant; *k*_e_ is the elimination rate constant equal to the clearance divided by the volume of distribution; *k*_p_ is the transfer rate constant between the central plasma compartment and the CSF compartment, which describes the rate of entry from the plasma to the CSF; PC is the partition coefficient, which represents that extent of drug that enters the CSF from the plasma. Image created in BioRender. Savic, R. (2025) https://BioRender.com/zr3xrpg.

The levels of linezolid in the plasma were best described by a 1-compartment model with linear absorption and clearance with both rate and extent to CSF estimated, with interindividual variability on clearance and on the plasma-to-CSF transfer rate constant. A schematic of the final model structure is depicted in Figure 1. Residual unexplained variability was described best by a combined additive and proportional model for plasma concentrations, and a proportional model for CSF concentrations. Covariate modeling resulted in an inclusion of effect of absolute rifampicin dose on linezolid clearance.

Participants receiving rifampicin at doses of 300, 450, 600, or 1200 mg were considered the reference group, and the impact of other dose levels on linezolid clearance was assessed, expressed as a percentage relative to the reference group. Participants receiving the highest doses, 1650 and 2100 mg, exhibited increased clearance, with an additional increase of 34.2% relative to the reference group. No statistically significant relationships could be identified for the remaining covariates assessed, including rifampicin dose on plasma-to-CSF rate or extent, age, sex, weight, baseline GCS, disease stage as measured by BMRC grade, route of medication administration, or baseline renal function.

**Figure 2.**
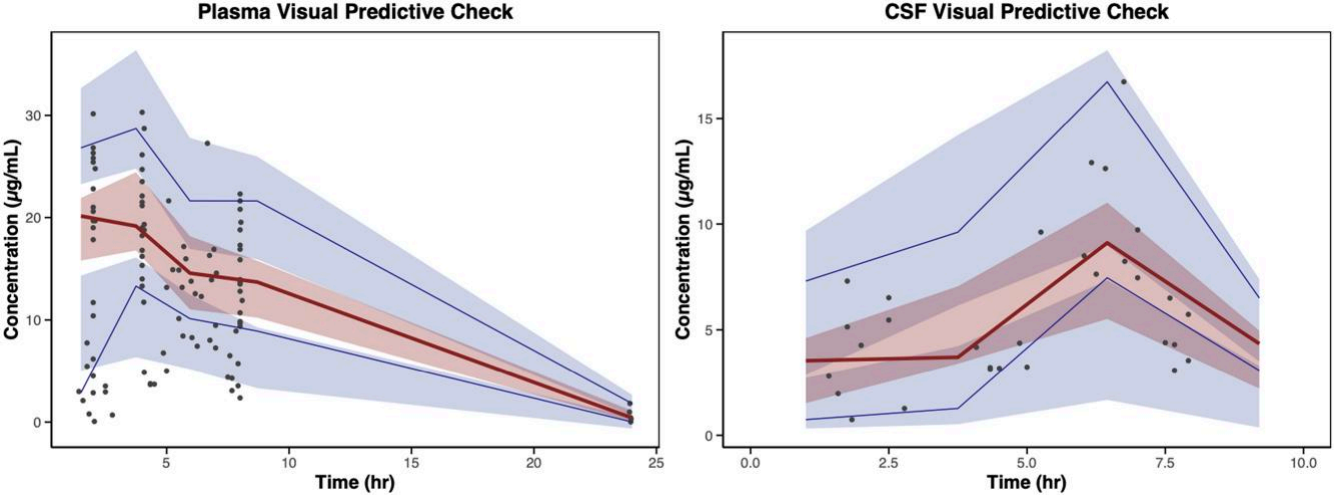
Visual predictive check (VPC) for the linezolid pharmacokinetic model in plasma and cerebrospinal fluid (CSF). The VPC compares observed and model-simulated concentrations over time. The black dots represent observed individual concentrations, while the blue-shaded areas depict the 95% confidence intervals (CIs) for the 5th and 95th percentiles of the simulated data. The red-shaded area represents the 95% CI for the median (50th percentile) of the simulated data. The solid dark lines correspond to the observed 5th (dark blue), 50th (red), and 95th (dark blue) percentiles of the data. The alignment between observed and simulated percentiles indicates the model's ability to capture the central trend and variability of drug exposure.

**Figure 3.**
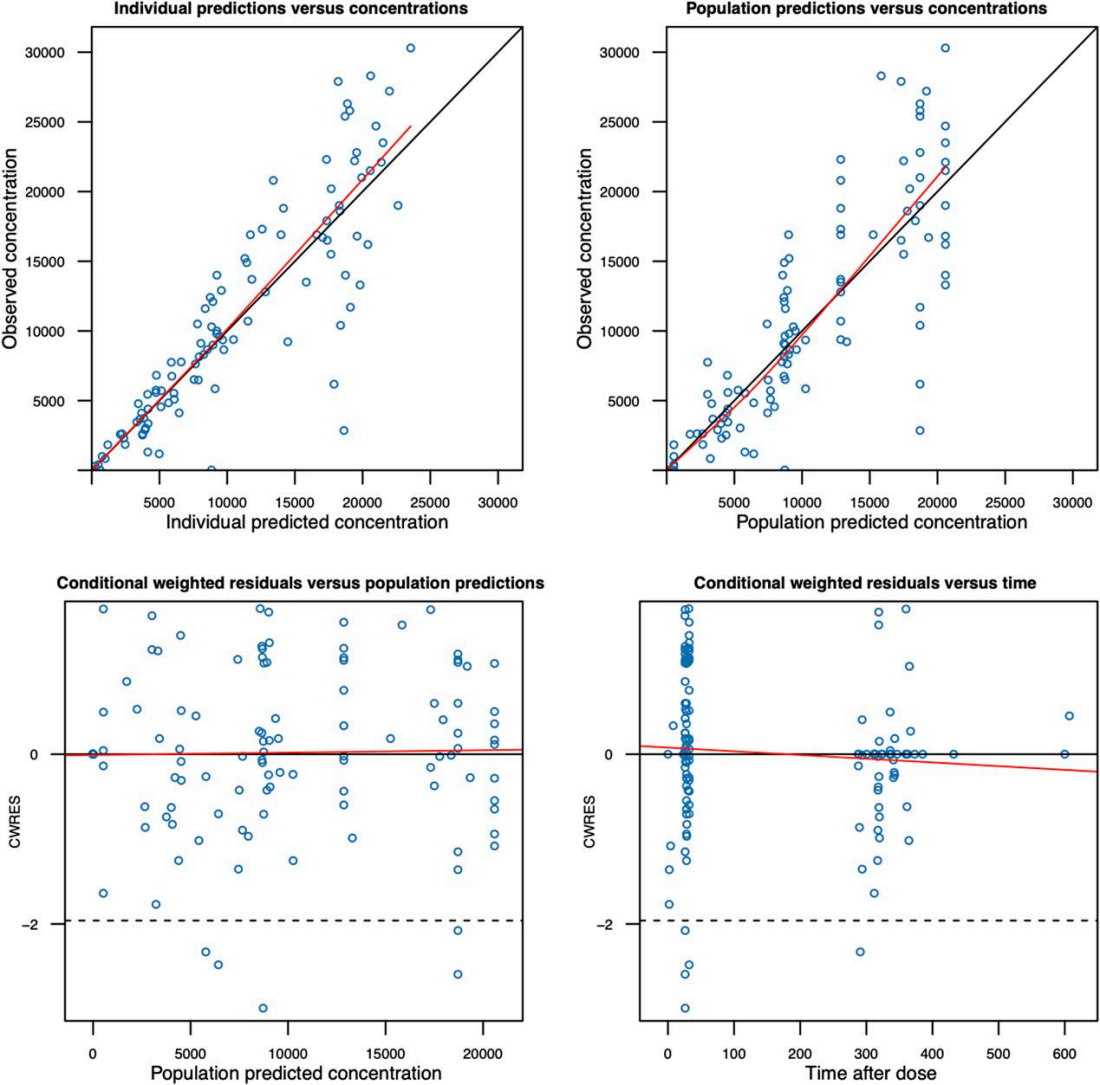
Goodness-of-fit diagnostic plots for linezolid pharmacokinetic model. Top left: observed versus individual-predicted concentrations—assesses how well the model predicts individual concentrations. Top right: observed versus population-predicted concentrations—evaluates model performance without accounting for interindividual variability. Bottom left: conditional weighted residuals versus population-predicted concentrations—checks for bias or trends in residuals across predicted values. Bottom right: conditional weighted residuals versus time—examines whether residuals show time-dependent patterns.

Once the plasma parameters were fixed and CSF concentrations were incorporated, the partition coefficient, representing the proportion of linezolid that entered the CSF from the plasma, was estimated at 53.8%. The rate of entry from plasma to CSF was estimated at 0.382 hour^−1^, with between-subject variability. The parameter estimates of the final PK model are presented in Table 2, and visual predictive checks and goodness-of-fit plots are represented in Figures 2 and 3, respectively.

**Table 2.** Parameter Estimates With Uncertainty of the Final Pharmacokinetic Model of Linezolid

Parameter	Estimate (95% CI) [RSE %]
CL: clearance, L/h	6.01 (5.01–7.01) [8.5]
*k*_a_: absorption rate constant, h^−1^	0.40 (.19–.61) [27.2]
*V*: volume of distribution, L	27.70 (16.27–38.77) [20.4]
*F*: relative bioavailability	1 (fixed)
*k*_p_: transfer rate constant between plasma and CSF, h^−1^	0.383 (.282–.484) [13.5]
PC: partition coefficient	0.535 (.458–.612) [7.4]
IIV on CL: interindividual variability in CL, %	17.7 [30.0]
IIV on *k*_p_: interindividual variability in *k*_p_, %	92.5 [24.7]
Plasma proportional error, %	33.3 [14.0]
Plasma additive error, µg/mL	0.26 [57.6]
CSF proportional error, %	31.6 [14.5]
Effect of 1650 or 2100 mg of rifampicin daily on CL (compared with lower doses), %	34.2 (2.4–66.4) [47.4]

Interindividual variability is expressed as percent coefficient of variation. Abbreviations: CSF, cerebrospinal fluid; RSE, relative standard error.

### Monte Carlo Simulations

**Figure 4.**
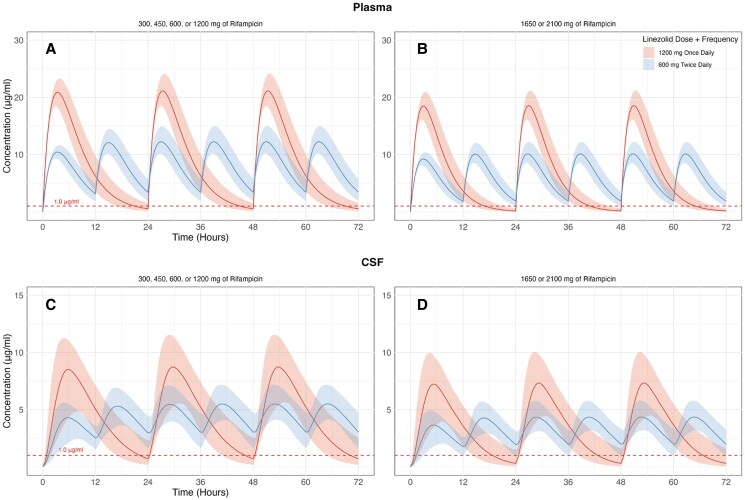
Monte Carlo simulated concentration–time profiles of linezolid in plasma (*A* and *B*) and cerebrospinal fluid (CSF) (*C* and *D*) for 2 dosing regimens and varying rifampicin doses. The red lines represent linezolid 1200 mg once daily, while the blue lines represent linezolid 600 mg twice daily. *A* and *C* illustrate linezolid concentrations with co-administration of either 300, 450, 600, or 1200 mg of rifampicin. *B* and *D* present linezolid concentrations with co-administration of either 1650 or 2100 mg of rifampicin. The red-dashed line at 1 μg/mL represents the minimum inhibitory concentration (MIC) of linezolid for *Mycobacterium tuberculosis*. Shaded areas indicate the simulated 95% confidence intervals for the concentration–time profiles.

Figure 4 illustrates the simulated concentration–time profiles of linezolid in plasma and CSF for typical trial participants, following either a once-daily dose of 1200 mg or a twice-daily dose of 600 mg. The profiles are presented for co-administration with varying doses of rifampicin. Panels A and C show the concentration–time profiles when co-administered with rifampicin doses of 300, 450, 600, or 1200 mg, while panels B and D reflect higher rifampicin doses of 1650 or 2100 mg. The red-dashed line indicates the MIC of 1 μg/mL for wild-type *M. tuberculosis.* The 95% confidence intervals for simulated concentrations are represented by shaded regions.

**Figure 5.**
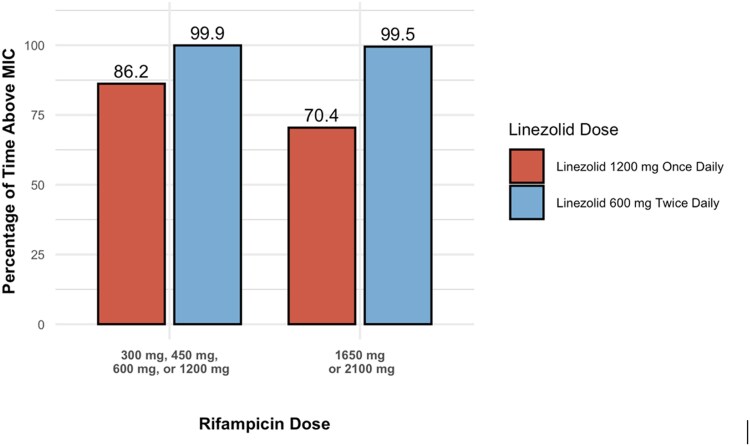
Percentage of time above the minimum inhibitory concentration (MIC) in plasma. The bar graph depicts the simulated percentage of time that linezolid plasma concentrations remain above an MIC of 1 μg/mL when co-administered with varying doses of rifampicin. Red bars correspond to a linezolid dosing regimen of 1200 mg once daily (QD), while blue bars represent a regimen of 600 mg twice daily (BID). Each bar reflects the impact of rifampicin dose on linezolid pharmacokinetics, highlighting differences in plasma exposure between the 2 linezolid dosing strategies.

**Figure 6.**
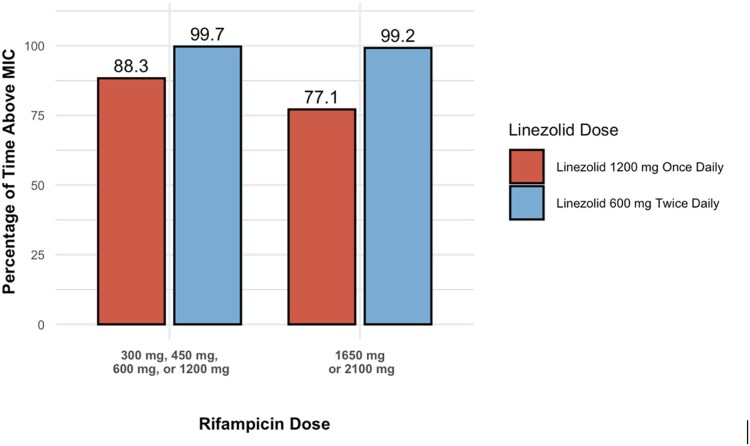
Percentage of time above the minimum inhibitory concentration (MIC) in cerebrospinal fluid (CSF). The bar graph depicts the simulated percentage of time that linezolid CSF concentrations remain above an MIC of 1 μg/mL when co-administered with varying doses of rifampicin. Red bars correspond to a linezolid dosing regimen of 1200 mg once daily (QD), while blue bars represent a regimen of 600 mg twice daily (BID). Each bar reflects the impact of rifampicin dose on linezolid pharmacokinetics, highlighting differences in plasma exposure between the 2 linezolid dosing strategies.

The percentage of time that plasma concentrations remained above the MIC (T > MIC) varied substantially based on both the linezolid dosing regimen and the rifampicin dose, as shown in Figure 5. When co-administered with lower doses of rifampicin (300–1200 mg), the once-daily 1200 mg regimen achieved a T > MIC of 86.2%, while the twice-daily 600 mg regimen demonstrated nearly complete coverage, with a T > MIC of 99.9%. T > MIC values for linezolid 1200 mg once daily decreased to 70.4% when co-administered with higher rifampicin doses (1650–2100 mg). In contrast, when co-administered with higher rifampicin doses, the 600 mg twice-daily regimen achieved consistent T > MIC levels of 99.5%, comparable to those observed at lower rifampicin doses. Similarly, Figure 6 depicts the percentage of time above the MIC in the CSF (CSF T > MIC) for the same regimens. At lower rifampicin doses, the 1200 mg once-daily regimen achieved 88.3% CSF T > MIC, while the 600 mg twice-daily regimen achieved 99.7%. At higher rifampicin doses, the once-daily regimen provided 77.1% CSF T > MIC compared to 99.2% for the twice-daily regimen.

## DISCUSSION

This study is the first to demonstrate that high-dose versus standard-dose rifampicin significantly impacts the extent of the rifampicin-linezolid drug–drug interaction in patients with TBM, as reflected by increased linezolid clearance but preserved plasma-to-CSF transfer. Our findings suggest that higher doses of rifampicin, such as those typical of 35 mg/kg dosing, increase linezolid clearance and reduce the time above the MIC. However, a dosing regimen of 600 mg twice daily, compared with 1200 mg once daily, may help mitigate these effects and maintain therapeutic drug levels. Ensuring sustained therapeutic concentrations is essential not only for optimizing treatment efficacy but also for preventing the emergence of bacterial resistance.

The PK of linezolid in plasma was effectively captured using a 1-compartment model characterized by first-order absorption and elimination. By linking CSF concentrations as levels within a hypothetical effect compartment, we were able to estimate both the rate and ratio of linezolid partitioning into the CSF from the plasma. Our findings suggest that the highest doses of rifampicin, those that are achieved with 35 mg/kg dosing compared with doses typical of standard 10 mg/kg dosing, enhance the clearance of linezolid from the systemic circulation. However, the processes governing the rate and extent of linezolid entry into the CSF appear unaffected by rifampicin co-administration. This observation suggests that rifampicin does not significantly modulate transporters or enzymatic pathways responsible for linezolid's CNS penetrance, such as P-glycoprotein, for example. Instead, the reduction in linezolid levels in the CNS at higher rifampicin doses is likely secondary to its impact on systemic clearance, leading to lower plasma concentrations and, subsequently, reduced availability for CNS partitioning. Similarly, no effect on linezolid absorption could be quantified, further suggesting no significant impact of high-dose rifampicin on P-glycoprotein transporters.

Despite these effects, twice-daily dosing of linezolid in conjunction with higher doses of rifampicin was shown to sustain CSF concentrations above 1 μg/mL, a target level associated with growth inhibition [15]. This finding underscores the importance of dose optimization when co-administering rifampicin to ensure adequate CNS exposure to linezolid, particularly in treating infections such as TBM, where CNS penetration is critical. The ALTER trial focused on the safety and tolerability of linezolid administered with high- and standard-dose rifampicin. Although the trial was not powered to detect a benefit in mortality or functional dependence, we observed a trend toward benefit of linezolid. If larger trials currently underway detect a clinical benefit of linezolid for the treatment of TBM, it will be imperative to optimize linezolid dosing and exposure to maximize benefit for patients with TBM.

Direct comparisons of our findings to other studies are influenced by differences in population characteristics, such as HIV status, disease severity, country of origin, ethnicity, dosing regimens, administration methods, rifampicin doses, and assay methodologies. Although our sampling design did not capture saturable kinetics observed in some studies, our clearance and volume estimates are comparable to those from other published models [17–20]. The Linezolid, Aspirin and Enhanced Dose Rifampicin in HIV-TBM (LASER-TBM) study, which evaluated the PK of linezolid in South African patients with HIV-associated TBM on high-dose rifampicin, reported PK parameter estimates consistent with ours, including maximal clearance, volume of distribution, the rate of CSF entry, and the CNS-to-plasma drug ratio for linezolid [21].

Our findings also align with other studies demonstrating a significant drug–drug interaction between linezolid and rifampicin [12, 13, 22–24]. While increased linezolid clearance has been observed when co-administered with rifampicin, with plasma concentrations reduced by up to 30% in healthy volunteers and even more in ill patients [22, 24–26], our study investigates the impact of high and standard-dose rifampicin on linezolid CSF concentrations, which is not well established. Linezolid undergoes predominantly nonenzymatic oxidative metabolism of the morpholine ring to form 2 inactive metabolites, which are eliminated primarily via renal excretion. Approximately one-third of administered linezolid is excreted unchanged in the urine, with the remainder recovered as inactive metabolites [27, 28]. Despite the absence of CYP involvement, rifampicin co-administration has been consistently associated with reduced linezolid exposure and increased apparent clearance. This interaction is therefore attributed to non-CYP mechanisms, which may include induction of drug transporters (eg, P-glycoprotein) affecting absorption and/or systemic clearance, enhancement of non-CYP oxidative pathways, and/or increased renal clearance. Although the precise mechanism has not been fully elucidated, the time-dependent nature of the interaction and its reproducibility across studies are consistent with rifampicin-mediated induction of clearance processes independent of CYP enzymes [29].

Several limitations in this study should be acknowledged. First, the small sample size, comprising only 18 participants from a single medical center in Masaka, Uganda, limits the generalizability and robustness of the findings. The modest number of participants receiving each rifampicin dose posed additional challenges. To address this, various rifampicin doses were treated as categorical variables. The effect of the median dose, along with the 2 closest doses, on linezolid clearance was considered as the baseline effect, while deviations in clearance at higher and lower doses were analyzed as percentages relative to this baseline. This approach compensated for the sparse data but precluded the analysis of rifampicin dose as a continuous variable or as separate categories for each dose. A larger sample size might have allowed for a more granular analysis of dose effects. The small number of participants and PK observations may also have impacted model performance, particularly with respect to the precision of estimates describing linezolid disposition in CSF. Moreover, the limited sample size likely constrained the power to reliably detect and quantify the influence of demographic and clinical covariates on linezolid PK variability.

Second, only sparse collection of CSF samples was available due to the invasive nature of LPs, limiting data density to 1 sample per participant per visit. However, randomizing the timing of CSF sample collection helped mitigate this limitation by spreading out the times at which each participant had their samples collected. This allowed a model-based approach to describe the full time-course of linezolid penetration into the CSF and should be considered in future sparse-sampling trials such as those that include CSF sampling.

Furthermore, the absence of a control arm without rifampicin precluded a comprehensive quantification of rifampicin's effect on linezolid clearance. Additionally, since maximal CYP3A4 induction occurs after ∼1 week, the timing of intensive PK sampling, which was performed on the day linezolid was initiated, limited the ability to fully capture induction effects. Sparse-sampling data were thus critical for informing the model on CYP3A4-mediated changes between rifampicin doses.

The heterogeneity in TBM diagnosis—5 definite, 12 probable, and 1 possible case—introduced potential variability due to disease–drug interactions. Unlike previous studies that used CSF protein levels to estimate a pseudo-partition coefficient, this study could not establish such correlations, and consequently, only free linezolid concentrations were analyzed. Finally, the therapeutic threshold concentration of 1 µg/mL was derived from in vitro data. While the World Health Organization has established a critical concentration of 1.0 µg/mL for linezolid susceptibility testing in wild-type *M. tuberculosis* using Middlebrook 7H10 agar, this value may not directly correspond to the concentrations required to achieve therapeutic efficacy in TBM. Further research is needed to validate this threshold in the context of TBM treatment.

Tuberculous meningitis is the most severe form of extrapulmonary TB, with high mortality and neurological complications. Treatment challenges stem from the inability of standard regimens—originally designed for pulmonary TB—to achieve sufficient drug concentrations in the CNS due to the restrictive BBB. While linezolid has demonstrated promising CNS penetration and bactericidal activity, its narrow therapeutic index and significant drug–drug interactions with rifampicin complicate its use in TBM treatment.

Our study demonstrates that very high-dose rifampicin regimens significantly increase linezolid systemic clearance and reduce plasma and CSF concentrations compared with standard-dose rifampicin regimens. However, twice-daily dosing of linezolid may mitigate these effects, maintaining CSF drug levels above the threshold required for bacterial inhibition. Notably, while rifampicin accelerates systemic clearance, it does not appear to directly impact the mechanisms governing linezolid's penetration into the CNS. These findings highlight the importance of optimizing linezolid dosing in TBM, ensuring effective CNS exposure while balancing potential drug–drug interactions, and the importance of model-based analysis to support these endeavors. Future studies with larger sample sizes and more intensive sampling strategies are needed to refine dosing recommendations and improve treatment outcomes for patients with TBM.

## Supplementary Material

ofag154_Supplementary_Data

## Supplementary Data

Supplementary materials are available at *Open Forum Infectious Diseases* online. Consisting of data provided by the authors to benefit the reader, the posted materials are not copyedited and are the sole responsibility of the authors, so questions or comments should be addressed to the corresponding author.

## References

[1] Marais S, Thwaites G, Schoeman JF, et al Tuberculous meningitis: a uniform case definition for use in clinical research. Lancet Infect Dis 2010; 10:803–12. 20822958 10.1016/S1473-3099(10)70138-9

[2] Agundez JA, Jiménez-Jiménez FJ, Alonso-Navarro H, García-Martín E. Drug and xenobiotic biotransformation in the blood-brain barrier: a neglected issue. Front Cell Neurosci 2014; 8:335. 25368552 10.3389/fncel.2014.00335 PMC4201098

[3] Dian S, Rahmadi R, van Laarhoven A, Ganiem AR, van Crevel R. Predicting mortality of tuberculous meningitis. Clin Infect Dis 2018; 67:1954–5. 29860408 10.1093/cid/ciy445

[4] Pardridge WM . Drug transport across the blood-brain barrier. J Cereb Blood Flow Metab 2012; 32:1959–72. 22929442 10.1038/jcbfm.2012.126 PMC3494002

[5] Dietze R, Hadad DJ, McGee B, et al Early and extended early bactericidal activity of linezolid in pulmonary tuberculosis. Am J Respir Crit Care Med 2008; 178:1180–5. 18787216 10.1164/rccm.200806-892OC PMC2588492

[6] Sun F, Ruan Q, Wang J, et al Linezolid manifests a rapid and dramatic therapeutic effect for patients with life-threatening tuberculous meningitis. Antimicrob Agents Chemother 2014; 58:6297–301. 25092692 10.1128/AAC.02784-14 PMC4187991

[7] Li H, Lu J, Liu J, Zhao Y, Ni X, Zhao S. Linezolid is associated with improved early outcomes of childhood tuberculous meningitis. Pediatr Infect Dis J 2016; 35:607–10. 26901441 10.1097/INF.0000000000001114

[8] Davis AG, Wasserman S, Maxebengula M, et al Study protocol for a phase 2A trial of the safety and tolerability of increased dose rifampicin and adjunctive linezolid, with or without aspirin, for HIV-associated tuberculous meningitis [LASER-TBM]. Wellcome Open Res 2021; 6:136. 34286103 10.12688/wellcomeopenres.16783.1 PMC8283551

[9] Chow FC, Kafeero P, Nakimbugwe M, et al Safety and tolerability of a short course of linezolid for the treatment of predominantly moderate to severe tuberculous meningitis in adults with human immunodeficiency virus. J Infect Dis 2025; 231:e1034–44. 39960851 10.1093/infdis/jiaf089 PMC12247810

[10] Chen J, Raymond K. Roles of rifampicin in drug-drug interactions: underlying molecular mechanisms involving the nuclear pregnane X receptor. Ann Clin Microbiol Antimicrob 2006; 5:3. 16480505 10.1186/1476-0711-5-3 PMC1395332

[11] Elmeliegy M, Vourvahis M, Guo C, Wang DD. Effect of P-glycoprotein (P-gp) inducers on exposure of P-gp substrates: review of clinical drug-drug interaction studies. Clin Pharmacokinet 2020; 59:699–714. 32052379 10.1007/s40262-020-00867-1 PMC7292822

[12] Alghamdi WA, Al-Shaer MH, An G, et al Population pharmacokinetics of linezolid in tuberculosis patients: dosing regimen simulation and target attainment analysis. Antimicrob Agents Chemother 2020; 64:e01174-20. 32778547 10.1128/AAC.01174-20 PMC7508612

[13] Hashimoto S, Honda K, Fujita K, et al Effect of coadministration of rifampicin on the pharmacokinetics of linezolid: clinical and animal studies. J Pharm Health Care Sci 2018; 4:27. 30459957 10.1186/s40780-018-0123-1 PMC6233381

[14] Strydom N, Ernest JP, Imperial M, et al Dose optimization of TBI-223 for enhanced therapeutic benefit compared to linezolid in antituberculosis regimen. Nat Commun 2024; 15:7311. 39181887 10.1038/s41467-024-50781-4 PMC11344811

[15] Bergstrand M, Hooker AC, Wallin JE, Karlsson MO. Prediction-corrected visual predictive checks for diagnosing nonlinear mixed-effects models. AAPS J 2011; 13:143–51. 21302010 10.1208/s12248-011-9255-z PMC3085712

[16] Lindbom L, Pihlgren P, Jonsson N. PsN-Toolkit—a collection of computer intensive statistical methods for non-linear mixed effect modeling using NONMEM. Comput Methods Programs Biomed 2005; 79:241–57. 16023764 10.1016/j.cmpb.2005.04.005

[17] Di Paolo A, Gori G, Tascini C, Danesi R, Del Tacca M. Clinical pharmacokinetics of antibacterials in cerebrospinal fluid. Clin Pharmacokinetics 2013; 52:511–42. 10.1007/s40262-013-0062-9 23605634

[18] Abdelgawad N, Wasserman S, Abdelwahab MT, et al Linezolid population pharmacokinetic model in plasma and cerebrospinal fluid among patients with tuberculosis meningitis. J Infect Dis 2024; 229:1200–8. 37740554 10.1093/infdis/jiad413 PMC11011161

[19] Imperial MZ, Nedelman JR, Conradie F, Savic RM. Proposed linezolid dosing strategies to minimize adverse events for treatment of extensively drug-resistant tuberculosis. Clin Infect Dis 2022; 74:1736–47. 34604901 10.1093/cid/ciab699 PMC9155613

[20] McGee B, Dietze R, Hadad DJ, et al Population pharmacokinetics of linezolid in adults with pulmonary tuberculosis. Antimicrob Agents Chemother 2009; 53:3981–4. 19564361 10.1128/AAC.01378-08 PMC2737850

[21] World Health Organization . Technical report on critical concentrations for drug susceptibility testing of medicines used in the treatment of drug-resistant tuberculosis. Geneva: WHO, 2018.

[22] Egle H, Trittler R, Kummerer K, Lemmen S. Linezolid and rifampin: drug interaction contrary to expectations? Clin Pharmacol Ther 2005; 77:451–3. 10.1016/j.clpt.2005.01.020 15900290

[23] Okazaki F, Tsuji Y, Seto Y, Ogami C, Yamamoto Y, To H. Effects of a rifampicin pre-treatment on linezolid pharmacokinetics. PLoS One 2019; 14:e0214037. 31518346 10.1371/journal.pone.0214037 PMC6743782

[24] Gandelman K, Zhu T, Fahmi OA, et al Unexpected effect of rifampin on the pharmacokinetics of linezolid: in silico and in vitro approaches to explain its mechanism. J Clin Pharmacol 2011; 51:229–36. 20371736 10.1177/0091270010366445

[25] Hoyo I, Martínez-Pastor J, Garcia-Ramiro S, et al Decreased serum linezolid concentrations in two patients receiving linezolid and rifampicin due to bone infections. Scand J Infect Dis 2012; 44:548–50. 22385321 10.3109/00365548.2012.663931

[26] Blassmann U, Roehr AC, Frey OR, et al Decreased linezolid serum concentrations in three critically ill patients: clinical case studies of a potential drug interaction between linezolid and rifampicin. Pharmacology 2016; 98:51–5. 27046487 10.1159/000445194

[27] Slatter JG, Stalker DJ, Feenstra KL, et al Pharmacokinetics, metabolism, and excretion of linezolid following an oral dose of [14C]linezolid to healthy human subjects. Drug Metab Dispos 2001; 29:1136–45. 11454733

[28] Stalker DJ, Jungbluth JJ. Clinical pharmacokinetics of linezolid, a novel oxazolidinone antibacterial. Clin Pharmacokinet 2003; 42:1129–40. 14531724 10.2165/00003088-200342130-00004

[29] Buerger C, Van Hasselt JGC, Schwartz F, et al Rifampicin reduces plasma concentrations of linezolid in patients. Antimicrob Agents Chemother 2006; 50:4315–7.

