# Supplementary material for: Impact of High-Dose Rifampicin on Linezolid Pharmacokinetics in Tuberculous Meningitis
Source: Open Forum Infect Dis. 2026 Mar 27;13(4):ofag154. doi: 10.1093/ofid/ofag154 (PMC13064523; doi:10.1093/ofid/ofag154)
Supplement: ofag154_Supplementary_Data [file ofag154_supplementary_data.docx]

Supplemental Table 1: Dosing weight bands


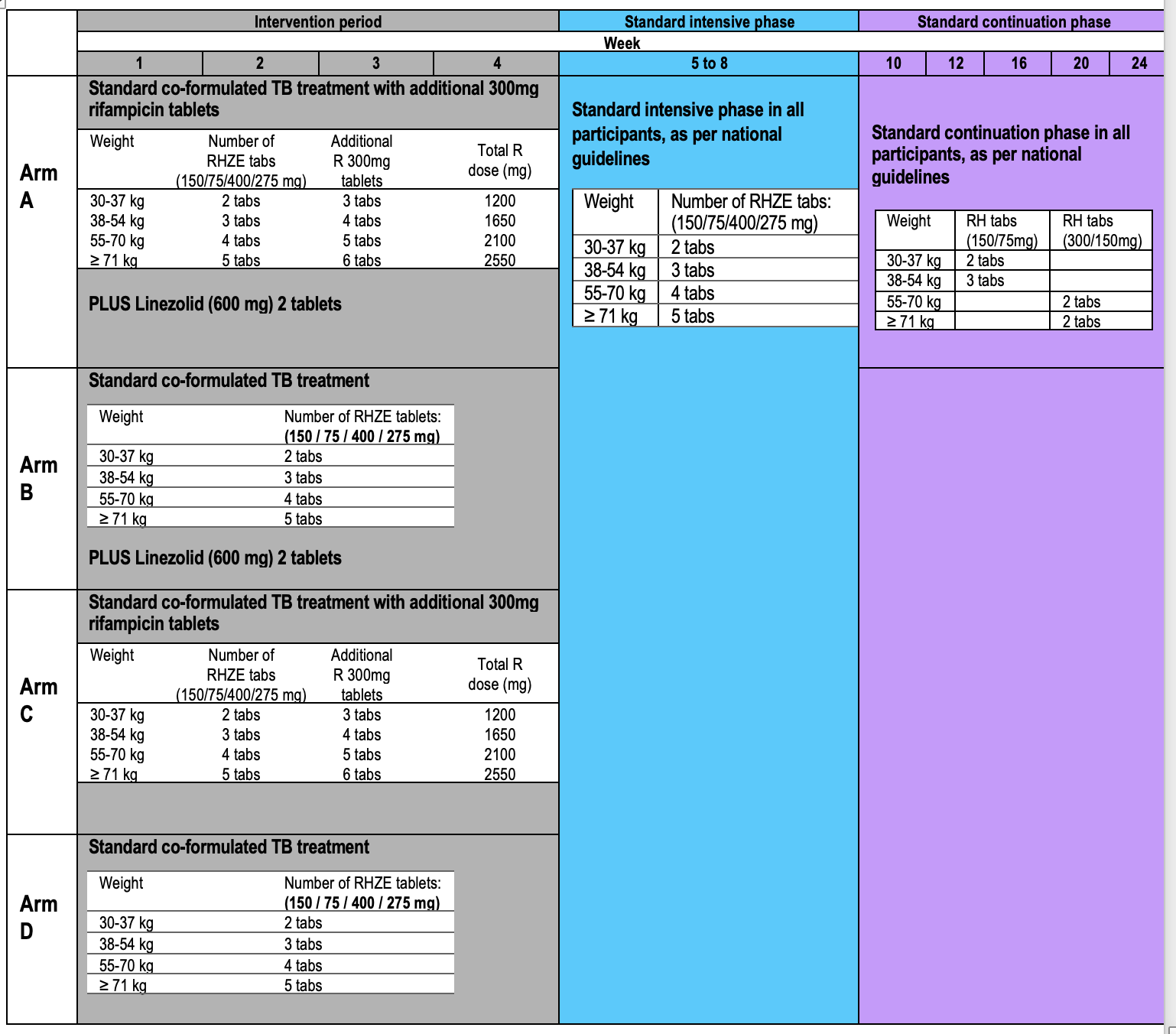


Supplemental Table 2: Plasma and CSF Linezolid Concentrations (ng/mL)

| Participant | Lumbar Puncture Timing* | Day 1 | | | | | Day 14 | |
| --- | --- | --- | --- | --- | --- | --- | --- | --- |
|  |  | Plasma | | | | CSF | Plasma | CSF |
|  |  | 0 hour | 2 hour | 4 hour | 8 hour |  |  |  |
| 1 | Early | BLQ | 11600 | 8150 | 4110 | 2585 | 4120 | 1855 |
| 2 | Early | BLQ | 10400 | 13300 | 13700 | 840 | 17900 | NA |
| 3 | Early | BLQ | 19000 | 16800 | 10700 | NA | NA | NA |
| 4 | Early | BLQ | 25400 | 21500 | 12800 | 7750 | NA | NA |
| 5 | Early | BLQ | 22800 | 19000 | 13500 | 5450 | 1830,  27200 | 2620 |
| 6 | Medium | BLQ | 10300 | 5850 | 1180 | 2300 | 7770 | 3035 |
| 7 | Medium | BLQ | 9110 | 6510 | 3460 | 2625 | 5090 | 2530 |
| 8 | Medium | BLQ | 21000 | 30300 | 22300 | 14900 | 28300 | 16900 |
| 9 | Medium | BLQ | 6180 | 14000 | 9380 | 4560 | NA | 6750 |
| 10 | Medium | BLQ | 2860 | 16200 | 17300 | 5710 | 411,  20200 | 1310 |
| 11 | Medium | BLQ | 27900 | 22200 | 15200 | 4400 | NA | NA |
| 12 | Late | BLQ | 26.2 | 12100 | 6820 | 3675 | 6480 | 2900 |
| 13 | Late | BLQ | 12400 | 8990 | 5580 | 4785 | 5530 | 3355 |
| 14 | Late | BLQ | 10000 | 9350 | 4840 | 3735 | 8660 | 5750 |
| 15 | Late | BLQ | 25800 | 22100 | 16900 | 14000 | 9220 | 9630 |
| 16 | Late | BLQ | 16500 | 15500 | 9800 | 10500 | 291,  16700 | NA |
| 17 | Late | BLQ | 11700 | 23500 | 18800 | 7630 | 996,  16900 | 8310 |
| 18 | Late | BLQ | 26300 | 24700 | 20800 | 12900 | 18.9,  18600 | 8650 |

BLQ, below limit of quantification; CSF, cerebrospinal fluid; NA, not available

*Early, 0-2 hours; medium, 2-6 hours; late, 6-8 hours
